# Identification of 4-Amino-7-chloroquinoline Derivative with In Vitro Activity Against Chikungunya Virus

**DOI:** 10.1007/s00284-026-05133-4

**Published:** 2026-08-22

**Authors:** Adriana Cotta Cardoso Reis, Camila Portruneli, Lívia da Cunha Agostini, Camila Rezende Minelli, Luana Neves Dias, Adalberto José de Lima, Breno de Mello Silva, Cíntia Lopes de Brito Magalhães, Glenda Nicioli da Silva, Guilherme Rocha Pereira, Geraldo Célio Brandão

**Affiliations:** 1 Pharmacy School, Department of Pharmacy, Federal University of Ouro Preto, Morro do Cruzeiro campus, Bauxita, Ouro Preto, 35402-163 MG Brazil; 2 Pharmacy School, Department of Clinical Analyses, Federal University of Ouro Preto, Ouro Preto, MG Brazil; 3 Department of Physics and Chemistry, Pontifical Catholic University of Minas Gerais, Belo Horizonte, MG Brazil; 4 Institute of Exact and Biological Sciences, Department of Biological Science, Federal University of Ouro Preto, Ouro Preto, MG Brazil

## Abstract

Arboviruses such as chikungunya virus (CHIKV), Zika virus (ZIKV), and Mayaro virus (MAYV) represent significant global health challenges due to the lack of effective antiviral therapies. Quinoline-based compounds have emerged as promising scaffolds for antiviral drug development. In this study, five 4-amino-7-chloroquinoline derivatives were synthesized and evaluated for their in vitro antiviral activity against CHIKV, ZIKV, and MAYV in Vero cells. Cytotoxicity and antiviral effects were assessed using a tetrazolium-based assay, followed by complementary analyses including cytopathic effect inhibition, virucidal assay, and viral load quantification by RT-qPCR. Among the tested compounds, derivative **6** demonstrated the most pronounced anti-CHIKV activity, with a low micromolar effective concentration and moderate selectivity index. This compound significantly reduced viral replication, preserved cell monolayer integrity, and decreased viral titers, achieving substantial viral load reduction confirmed by RT-qPCR. No virucidal activity was observed. In contrast, compound **6** inhibited the virus-induced cytopathic effect when administered after viral adsorption, suggesting that its antiviral activity is unlikely to result from interference with viral attachment or adsorption but rather from inhibition of a post-adsorption stage of the viral replication cycle. Overall, these findings highlight 4-amino-7-chloroquinoline derivatives as a promising class of antiviral agents and identify compound **6** as a potential lead candidate for further development against CHIKV.

**Supplementary Information:**

The online version contains supplementary material available at https://doi.org/10.1007/s00284-026-05133-4.

## Introduction

Arthropod-borne viruses (arboviruses) constitute a major group of emerging and re-emerging pathogens transmitted primarily by mosquitoes of the *Aedes* and *Culex* genera [1]. These viruses are widely distributed in tropical and subtropical regions and have become an increasing global public health concern due to their expanding geographic range and rising incidence [2]. Among them, dengue virus (DENV), Zika virus (ZIKV), chikungunya virus (CHIKV), and Mayaro virus (MAYV) are of particular relevance, as they are responsible for large outbreaks associated with fever, rash, and severe musculoskeletal symptoms, often leading to long-term morbidity [3–5].

Despite their clinical and epidemiological importance, there are currently no specific antiviral therapies approved for most arboviral infections, and treatment remains largely supportive [6–8]. The continued emergence and co-circulation of these viruses highlight the urgent need to identify and develop novel antiviral agents with improved efficacy and safety profiles. In this context, small synthetic molecules have played a central role in drug discovery, offering structural diversity and opportunities for rational optimization [6–8].

Quinoline and its derivatives represent a privileged scaffold in medicinal chemistry, exhibiting a broad spectrum of biological activities, including well documented antiviral effects [9]. Structural modifications of the quinoline core have led to compounds with activity against several RNA viruses, supporting their potential as templates for the development of new antiviral agents. In particular, 4-aminoquinoline derivatives have attracted attention due to their pharmacological versatility and favorable drug like properties.

In the present study, we report the synthesis and in vitro evaluation of novel 4-amino-7-chloroquinoline derivatives against CHIKV, ZIKV, and MAYV. Furthermore, mechanistic insights into the antiviral activity of the most active compound were investigated through cytopathic effect inhibition assays, virucidal assays, and viral load quantification by reverse transcription-quantitative polymerase chain reaction (RT-qPCR). This work aims to identify promising lead compounds and contribute to the development of new therapeutic strategies against medically relevant arboviruses.

## Materials and Methods

### Chemicals and Synthesis of Compounds

**Fig. 1:**
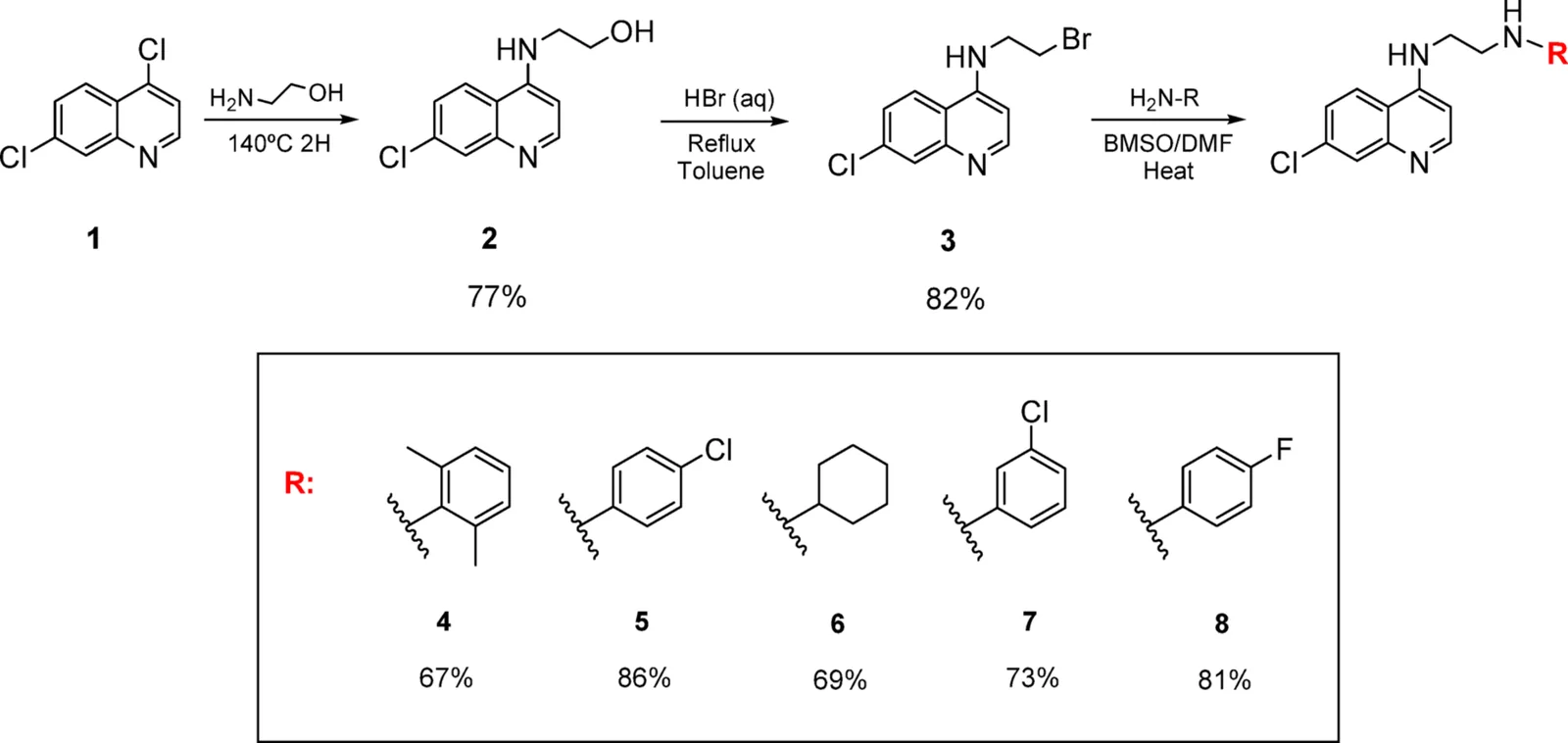
Synthetic route scheme of the 4-amino-7-chloroquinoline derivatives

All reagents and solvents were of analytical grade and used without further purification unless otherwise stated. The 4-amino-7-chloroquinoline derivatives were synthesized through a three-step synthetic route. Structural characterization of the compounds was performed by ^1^H and ^13^C nuclear magnetic resonance (NMR) spectroscopy and high-resolution mass spectrometry (HRMS). Detailed synthetic procedures and spectral data are provided in the Supplementary Information. The synthetic route is summarized in Fig. 1.

### Cell Lines and Viruses

Vero cells (ATCC CCL-81) were maintained in Dulbecco’s Modified Eagle Medium (DMEM, high glucose) supplemented with 5% fetal bovine serum (FBS) and antibiotics under standard conditions (37 °C, 5% CO₂). The mosquito-derived C6/36 cell line was cultured in Leibovitz’s L-15 medium supplemented with 5% FBS.

Zika virus (ZIKV, strain PE243/2015) was propagated in C6/36 cells. Chikungunya virus (CHIKV, strain 27-African) and Mayaro virus (MAYV, strain Acre27) were propagated in Vero cells. Viral stocks were obtained from infected cell culture supernatants, clarified by centrifugation, aliquoted, and stored at − 80 °C. Viral titers were determined prior to use.

### Cytotoxicity Assay

Cell viability was evaluated using the MTT assay. Vero cells (1 × 10⁴ cells/well) were seeded in 96-well plates and incubated with serial dilutions of the synthesized compounds (0.78–100 µg mL⁻¹) prepared in dimethyl sulfoxide (DMSO). After incubation, MTT reagent was added and absorbance was measured spectrophotometrically. The median cytotoxic concentration (CC₅₀) was determined from dose response curves [10, 11].

### In Vitro Antiviral Assay

Antiviral activity was assessed using an MTT-based assay in Vero cells (1 × 10⁴ cells/well) infected with CHIKV, ZIKV, or MAYV at defined viral titers. Cells were treated with non-cytotoxic concentrations of the compounds [12, 13]. The median effective concentration (EC₅₀), defined as the concentration required to protect 50% of infected cells from virus-induced cytopathic effects, was calculated [14]. The selectivity index (SI) was determined as the ratio CC₅₀/EC₅₀. Amantadine and chloroquine were used as positive controls for CHIKV, while ribavirin was used for ZIKV and MAYV [15].

### Virucidal Assay

The virucidal activity of the most active compound was evaluated by pre-incubating the compound (6 µM) with CHIKV prior to infection. After incubation, residual infectivity was determined using a plaque reduction assay, and viral titers were expressed as plaque forming units per milliliter (PFU mL⁻¹) [15–17].

### Inhibitory Cytopathic Effect Assay

The inhibitory effect on virus induced cytopathic effects (CPE) was assessed in Vero cells (1 × 10⁶ cells/well) seeded in 6-well plates. Cells were infected with CHIKV (MOI = 1) and treated with the selected compound (6 µM). After 48 h, cell monolayers were examined microscopically to evaluate morphological changes [18]. Supernatants were collected for viral titration by plaque assay.

### Viral Load Quantification by RT-qPCR

Viral RNA was extracted from supernatants using a commercial extraction kit according to the manufacturer’s instructions. Complementary DNA (cDNA) was synthesized using a reverse transcription kit. Quantitative PCR (RT-qPCR) was performed using SYBR Green chemistry to determine viral RNA levels at 24, 36, and 48 h post-infection.

Relative viral quantification was based on standard curves generated from serial dilutions of known viral titers. Results were expressed as log₁₀ PFU mL⁻¹ equivalents.

### Viral RNA Extraction

Viral RNA was extracted using the Bio Gene Viral DNA/RNA Extraction kit (Bioclin/Quibasa) following the manufacturer’s instructions. The quality and quantity of the extracted RNA were assessed by measuring the A₂₆₀/₂₈₀ and A₂₆₀/₂₃₀ ratios, as well as the concentration (ng µL⁻¹), using a NanoDrop™ Lite Plus spectrophotometer (Thermo Fisher), according to Loaiza-Cano et al. with modifications [19].

### cDNA Synthesis

Complementary DNA (cDNA) was obtained from the High-Capacity cDNA Reverse Transcription Kit (Applied Biosystems, Waltham, Massachusetts, USA) according to Monsalve-Escudero et al. and Souza et al., with modifications [20, 21]. The amplification program consisted of a cycle of 25 °C for 10 min, 37 °C for 120 min, 85 °C for 5 min and holding at 4 °C, following the manufacturer’s recommendations.

### RT-qPCR

For the relative quantification of CHIKV viral RNA, quantitative PCR (RT-qPCR) was performed using GoTaq^®^ qPCR Master Mix kit (Promega) containing SYBR™ Green, according to Cuellar-Quimbaya et al., with modifications [22]. To estimate the relative concentration of viral genetic material, serial dilutions with a dilution factor of 1:10 were performed. Six points were generated in duplicates on the standard curve with 1 × 10⁹, 1 × 10⁸, 1 × 10⁷, 1 × 10⁶, 1 × 10⁵, and 1 × 10⁴ PFU mL⁻¹ equivalents. The primer sequences for CHIKVF were 5’-GACAATGCGCGCGGTACC-3’ and CHIKVR were 5’-TGTTGTTTTGTGGCGCCT-3’^40^. The amplification program consisted of a 95 °C hold cycle for 2 min and 40 cycles of 95 °C for 15 s, 51 °C for 15 s, and 72 °C for 1 min. The melting curve was standardized at 95 °C for 15 s, 60 °C for 1 min, 95 °C for 30 s (dissociation), and 60 °C for 15 s.

The relative quantification of the viral load in the sample was performed by analyzing the quantification cycle (Cq). The Cq value was inserted into a straight-line equation constructed from a standard curve generated with known concentrations of the CHIKV genome (converted to Log₁₀ PFU mL⁻¹). Subsequently, the result was compared to the log₁₀ PFU mL⁻¹ values of the virus control samples (VC), allowing the determination of the relative viral load of CHIKV [22–24].

### Statistical Analysis

All experiments were performed in at least duplicate or triplicate. Data were expressed as mean ± standard deviation (SD). Statistical analyses were conducted using Student’s *t*-test, and differences were considered statistically significant at *p* ≤ 0.05.

## Results

### Synthesis and Characterization of Compounds

Five 4-amino-7-chloroquinoline derivatives were successfully synthesized with yields ranging from 67 to 86%. Structural characterization by ^1^H and ^13^C NMR spectroscopy and high-resolution mass spectrometry (HRMS) confirmed the expected chemical structures. The spectral data were consistent with the proposed quinoline framework and corresponding substituents. Detailed characterization data are provided in the Supplementary Information.

### Cytotoxicity and Antiviral Activity

The cytotoxicity of the synthesized compounds was initially evaluated in Vero cells to determine CC₅₀ values and define non-toxic concentrations for antiviral assays. The compounds exhibited CC₅₀ values ranging from 13.3 to 98.1 µM (Table 1).

**Table 1:** In vitro cytotoxicity (CC_50_) and antiviral activity (EC_50_) assessments of chloroquinoline derivatives against CHIKV, MAYV, and ZIKV in Vero cells. The results include standard deviations (n = 3), selectivity index (SI), and relative potency (RP) values, showcasing the compounds' potential antiviral and cytotoxic profiles.


SUBSTANCE	R	VERO CC_50_(µM)	ZIKV ^a^ EC_50_(µM)	SI^b^	RP_R_^c^	RP_C_^d^	CHIKV^e^ EC_50_(µM)	SI	RP_A_^f^	RP_C_	MAYV^g^ EC_50_(µM)	SI	RP_R_	RP_C_
**4**		30.5 ± 1.3	19.4 ± 1.8	1.6	68.4	3.7	NA^h^	–^i^	–	–	25.83 ± 1.56	1.2	51.2	1.6
**5**		13.3 ± 1.4	NA	–	–	–	NA	–	–	–	NA	–	–	–
**6 **		21.0 ± 1.4	NA	–	–	–	4.9 ± 2.6	4.26	279.69	7.4	NA	–	–	–
**7**		81.7 ± 1.2	NA	–	–	–	NA	–	–	–	NA	–	–	–
**8**		98.1 ± 1.3	NA	–	–	–	NA	–	–	–	NA	–	–	–
**Chloroquine ** **(Positive control)**		131.1 ± 1.3	72.5 ± 3.5	1.8	–	–	36.4 ± 1.6	3.6	–	–	83.4 ± 2.3	1.6	–	–
**Ribavirin (Positive control)**	–	1516.7 ± 1.2	433.2 ± 1.6	3.5	–	–	NT	–	–	–	431.2 ± 2.2	3.5	–	–
**Amantadine** **(Positive control)**	–	561.3 ± 1.2	NT^j^	–	–	–	419.0 ± 1.7	1.3	–	–	NT	–	–	–

^a^ ZIKV: viral titre 1x10^3^ TCID_50_.mL^− 1^; ^b^ SI (Selectivity Index): the ratio of CC_50_ to EC_50_; ^c^ RP_**R**_ (Relative Potency): ratio between Ribavirin positive control EC_50_ and active compound EC_50_; ^d^ RP_**C**_ (Relative Potency): ratio between Chloroquine positive control EC_50_ and active compound EC_50_; ^e^ CHIKV: viral titre 1x10^4^ TCID_50_.mL^− 1^; ^f^ RP_**A**_ (Relative Potency): ratio between Amantadine positive control EC_50_ and active compound EC_50_; ^g^ MAYV: viral titre 1x10^4^ TCID_50_.mL^− 1^; ^h^ NA: Not active; ^i^ –: Not determined, ^j^ NT: Not tested

**Fig. 2:**
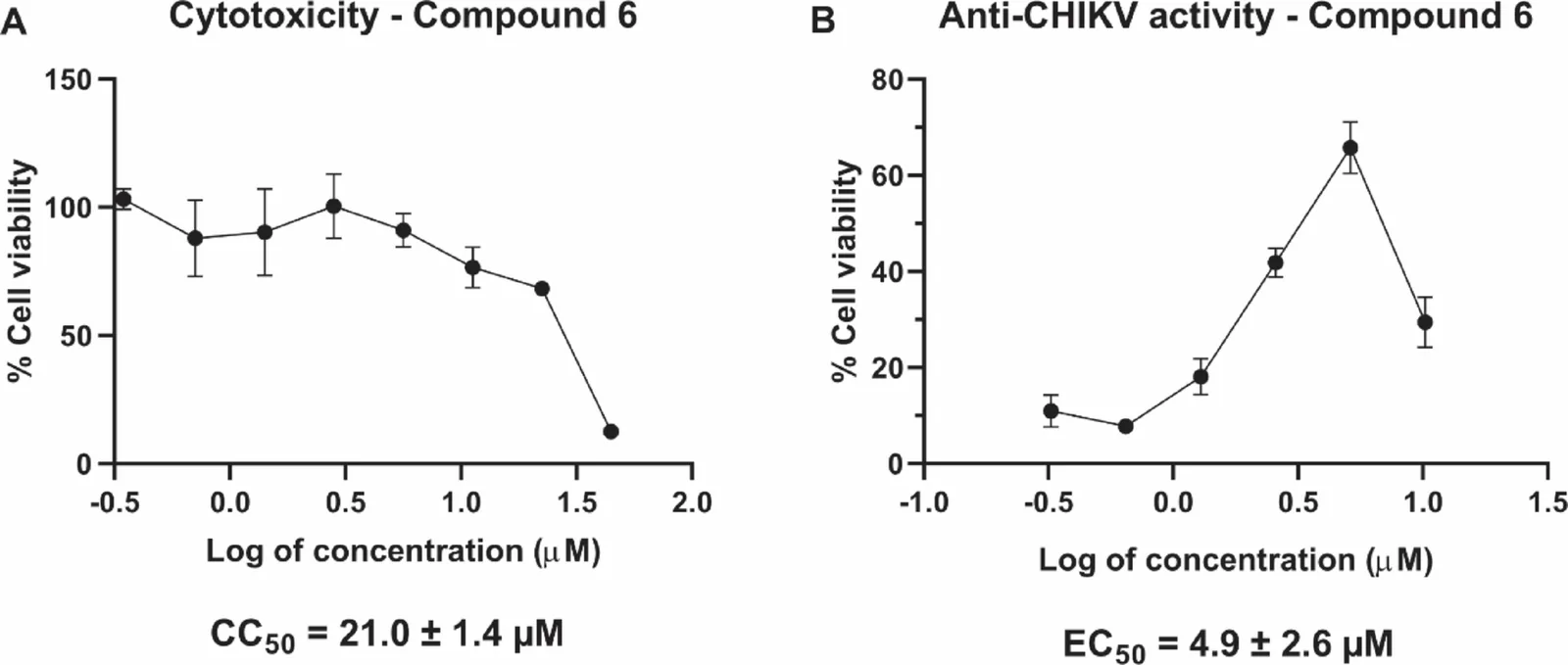
Dose–response curves of chloroquinoline derivative **6** showing (**a**) cytotoxicity (CC₅₀) and (**b**) antiviral activity (EC₅₀) against CHIKV in Vero cells, as determined by the MTT assay after 72 h of treatment (*n* = 3). Data are presented as the mean ± standard deviation of cell viability relative to untreated cell control

Among the tested derivatives, two compounds demonstrated antiviral activity. Compound **4** showed moderate activity against ZIKV and MAYV, with EC₅₀ values of 19.4 µM and 25.8 µM, respectively, but low selectivity indices. In contrast, compound **6** exhibited selective antiviral activity against CHIKV, with an EC₅₀ of 4.9 µM and a selectivity index of 4.26 (Table 1; Fig. 2). No significant antiviral activity was observed for the remaining derivatives.

### Virucidal Activity

The virucidal assay was performed to evaluate whether compound **6** directly inactivates viral particles prior to cell infection. Pre-incubation of CHIKV with compound **6** (6 µM) did not result in a significant reduction in viral titers compared to the viral control (Table 2), indicating the absence of virucidal activity under the tested conditions.

**Table 2:** Quantification of viruses (PFU.mL⁻¹) after virucidal effect assay (*n* = 2) of compound **6** (6 µM) against CHIKV (MOI = 10)

	Titration media (PFU.mL^− 1^)	Log_10_ PFU.mL^− 1^
CHIKV (viral control)	1.51 × 10^5^	5.16
**6 + CHIKV**	1.02 × 10^5^	5.01

CHIKV (virus control suspension), 6 + CHIKV [(Compound 6 (6 µM) was incubated with CHIKV particles suspension]

### Inhibition of Cytopathic Effects

**Fig. 3:**
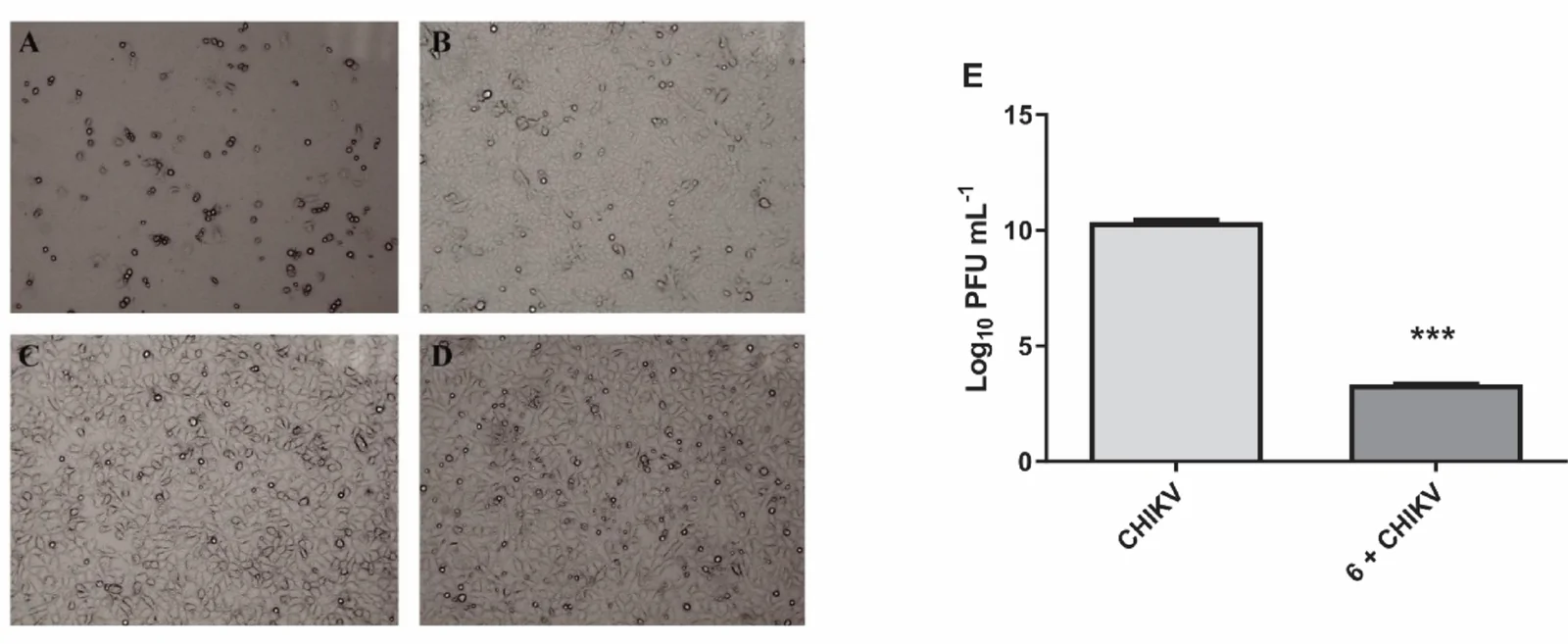
Inhibitory effect of synthesized compound 6 (6 µM) on CHIKV-induced cytopathic effect and viral production in Vero cells. Representative images of Vero cell monolayers obtained 48 h post-infection with CHIKV (MOI = 1) under the following conditions: (**a**) viral control, infected and untreated cells; (**b**) cell control, uninfected and untreated cells; (**c**) cells infected with CHIKV and treated with compound **6** (6 µM); and (**d**) cytotoxicity control, uninfected cells treated with compound **6** (6 µM). Images were acquired under 100× magnification using ZEN 2.3 Lite imaging software. (**e**) Viral yield quantification by plaque assay of supernatants collected from CHIKV-infected Vero cells following treatment with compound **6** (6 µM) or no treatment (viral control). CHIKV: supernatant from infected and untreated monolayers; 6 + CHIKV: supernatant from infected monolayers treated with compound **6** (6 µM). Experiments were performed in duplicate, and statistical significance was assessed using Student’s *t*-test with a 95% confidence interval. Data are presented as mean ± standard deviation. **p < 0.0001

The antiviral activity of compound **6** was further assessed by its ability to inhibit CHIKV-induced cytopathic effects (CPE) in Vero cells. At 48 h post-infection, untreated infected cells exhibited pronounced morphological alterations, including cell rounding and detachment. In contrast, infected cells treated with compound **6** (6 µM) showed preservation of the cell monolayer and reduced cytopathic damage (Fig. 3A-D).

Viral titration of the supernatants revealed a marked reduction in viral production in treated cultures. Specifically, a decrease of approximately 7 log₁₀ PFU mL⁻¹ was observed compared to the viral control (Table 3; Fig. 3E).

**Table 3:** Quantification of viral titers (PFU.mL⁻¹) following the inhibitory CPE assay of compound **6** (6 µM) against CHIKV (MOI = 1), performed in duplicate

	Titration media (PFU.mL^− 1^)	Log_10_ PFU.mL^− 1^
CHIKV viral control	2.30 × 10^10^	10.36
**6 + CHIKV**	2.15 × 10^3^	3.33

CHIKV: viral control (supernatant of the infected monolayer); 6 + CHIKV: supernatant of the infected monolayer treated with compound **6** (6 µM)

**Fig. 4:**
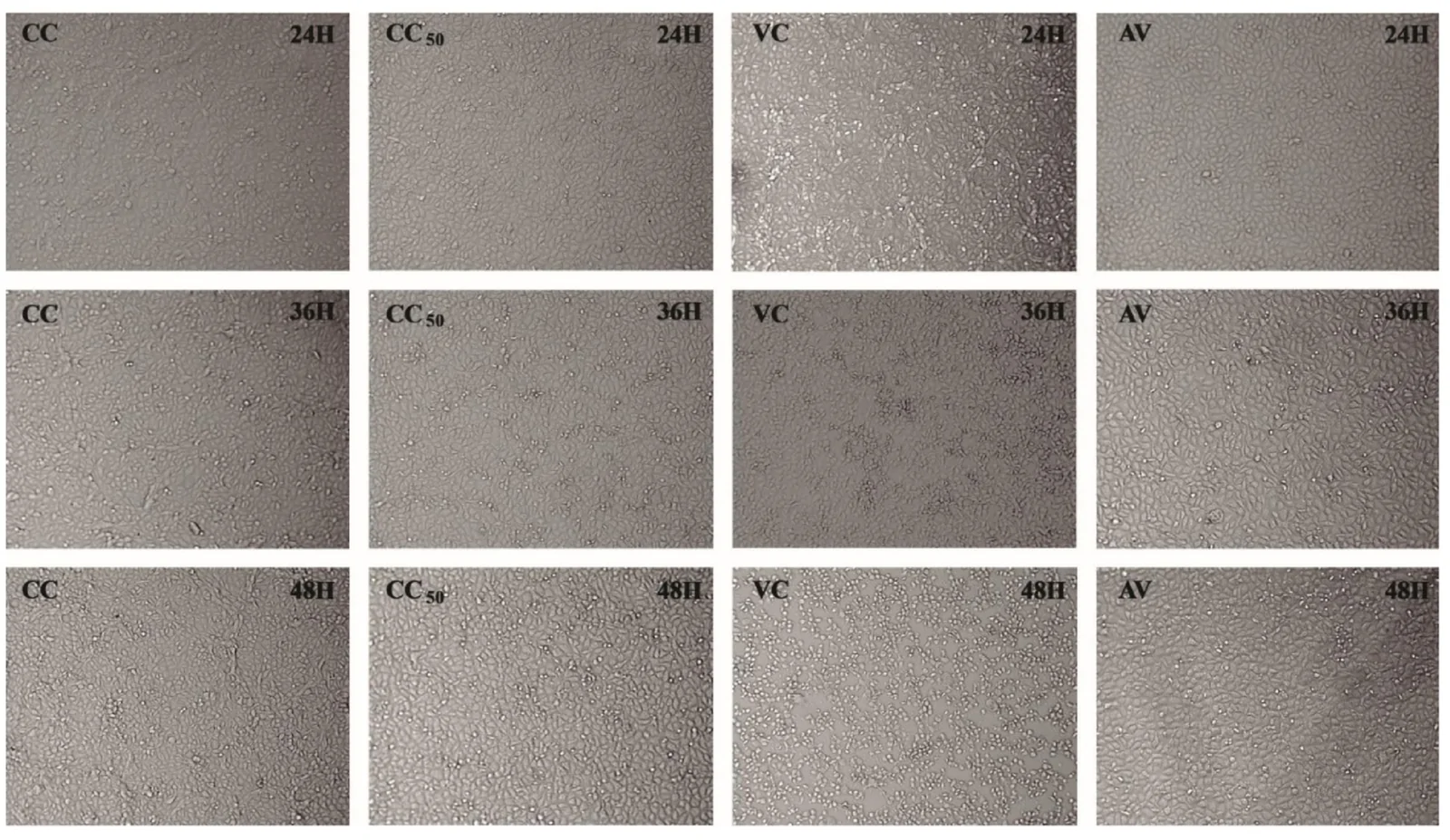
Inhibitory effect of synthesized compound **6** (6 µM) on CHIKV-induced cytopathic effect (CPE) in Vero cells. The analysis included uninfected cells, compound **6** cytotoxicity, and virus infected controls. CPE inhibition was evaluated at 24-, 36-, and 48-hours post-infection (MOI = 1). Representative images were obtained using ZEN 2.3 Lite software at 100× magnification. CC: Cell Control, CC_50_: Cytotoxicity Control (6 µM), VC: viral control, AV: antiviral activity of compound **6** (6 µM)

The active compound **6** (6 µM) was investigated via RT-qPCR to validate its antiviral activity against CHIKV, as observed in the MTT and inhibitory CPE assays. Viral genetic material was quantified, comparing treated and infected Vero cells with viral control at 24-, 36-, and 48-hour post-infection. The results were supported by photographs of cell monolayers, which displayed a clear reduction in viral replication, as evidenced by the preservation of cell structure (Fig. 4).

### Viral Load Reduction by RT-qPCR

**Fig. 5:**
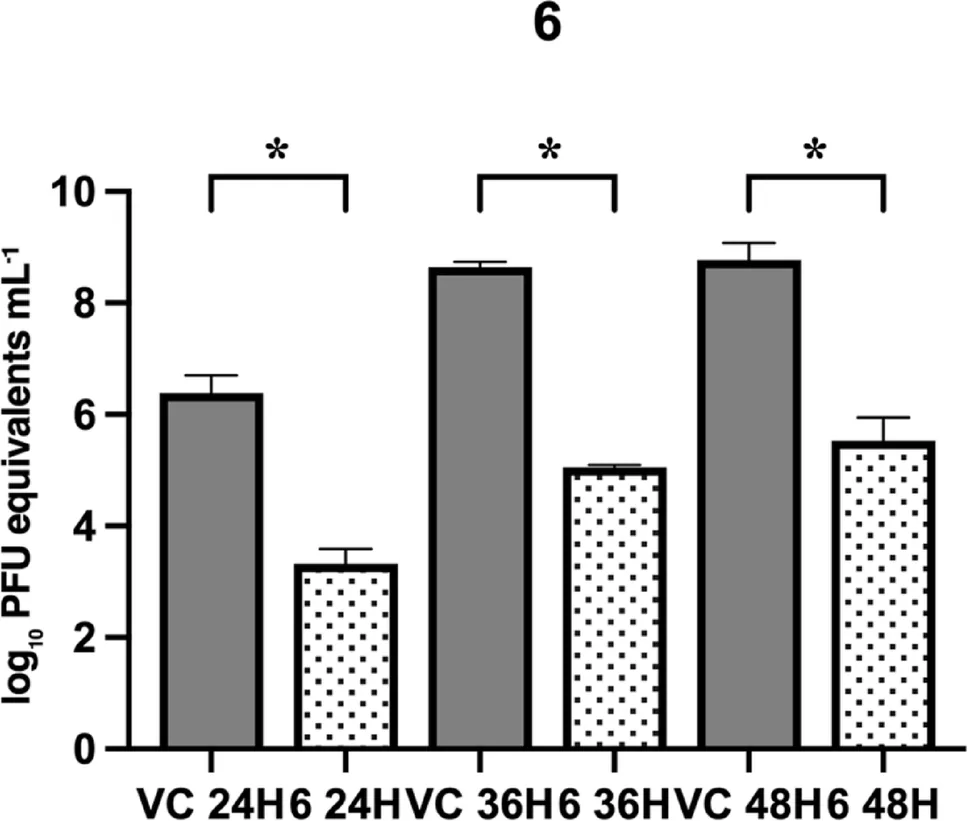
Viral quantification of CHIKV following the inhibitory cytopathic effect (CPE) assay with compound **6** (6 µM) at 24-, 36-, and 48-hours post-infection (MOI = 1) in Vero cells (*n* = 3). Viral load was measured by RT-qPCR and expressed as log₁₀ PFU equivalents mL⁻¹ based on a standard curve generated from known CHIKV concentrations. Viral load was measured by RT-qPCR, and statistical analysis was performed using Student’s t-test with a 95% confidence interval. VC: virus control; **6** 24 H: anti-CHIKV activity of compound **6** (6 µM) 24 h post-infection; **6** 36 H: anti-CHIKV of compound **6** (6 µM) 36 h post-infection; **6** 48 H: anti-CHIKV of compound **6** (6 µM) 48 h post infection. Error bars represent standard deviation; *: p value < 0.05

The effect of compound **6** on viral replication was further quantified by RT-qPCR. Treatment of CHIKV-infected Vero cells resulted in a significant reduction in viral RNA levels (detected in cell culture supernatants), expressed as log₁₀ PFU equivalents mL⁻¹, at 24, 36, and 48 h post-infection compared to untreated controls (Fig. 5). The decrease in viral load was consistent with the results obtained from the cytopathic effect inhibition and plaque reduction assays.

## Discussion

Arboviruses such as CHIKV, ZIKV, and MAYV continue to pose significant public health challenges, particularly in tropical and subtropical regions, due to their rapid spread and the absence of specific antiviral therapies [5, 25]. In this context, the identification of novel small molecules with antiviral activity remains a priority.

In the present study, a series of 4-amino-7-chloroquinoline derivatives was synthesized and evaluated for in vitro antiviral activity. Among the tested compounds, derivative **6** demonstrated selective and potent activity against CHIKV, with a low micromolar EC₅₀ and a selectivity index above the threshold commonly considered acceptable for antiviral candidates [26]. In contrast, compound **4** exhibited only moderate activity against ZIKV and MAYV, with limited selectivity, suggesting a narrower therapeutic potential.

The observed differences in antiviral activity among the derivatives highlight the influence of structural modifications on biological activity. In particular, the presence of a cyclohexyl moiety in compound **6** appears to be associated with enhanced anti-CHIKV activity, whereas other substituents, including halogenated phenyl groups, did not confer significant antiviral effects. These findings reinforce the importance of substituent optimization in quinoline based scaffolds for antiviral drug development.

The absence of virucidal activity indicates that the antiviral effect of compound **6** is unlikely to result from the direct inactivation of viral particles. Instead, the available evidence suggests that derivative **6** interferes with a post-adsorption stage of the viral replication cycle. This interpretation is supported by the cytopathic effect inhibition assay, in which treatment initiated after viral adsorption preserved cell monolayer integrity, together with the marked reduction in viral titers observed in plaque assays. Although these findings are consistent with inhibition of post-adsorption events, additional mechanistic studies are required to identify the precise molecular target and stage of the viral replication cycle affected by the compound.

Furthermore, RT-qPCR analysis confirmed a significant decrease in CHIKV RNA levels in treated cells at different time points post infection, demonstrating that compound **6** effectively suppresses viral replication. These findings are consistent with previous studies reporting antiviral activity of quinoline derivatives against RNA viruses, including CHIKV, and suggest that such compounds may act by targeting viral nonstructural proteins or host dependent pathways involved in replication [27, 28].

Previous reports have indicated that quinoline based compounds may interfere with key viral processes, including endosomal acidification and viral protein function [26]. In the case of CHIKV, potential targets include the nsP2 and nsP3 nonstructural proteins, as well as envelope glycoproteins involved in viral entry [29]. Although the precise mechanism of action of compound **6** was not elucidated in this study, the lack of virucidal effect combined with the observed reduction in intracellular viral RNA supports a post entry mode of action.

Overall, the results demonstrate that 4-amino-7-chloroquinoline derivatives represent a promising class of antiviral agents. In particular, compound **6** emerges as a potential lead candidate for the development of new therapeutics against CHIKV. Further studies, including mechanistic investigations and in vivo evaluations, are warranted to fully characterize its antiviral potential and optimize its pharmacological profile.

### Conclusion and Future Directions

In summary, the present study demonstrates that 4-amino-7-chloroquinoline derivatives show antiviral potential against arboviruses. Among the synthesized molecules, compound **6** exhibited selective and potent in vitro activity against CHIKV, significantly reducing viral replication and preserving cell integrity without displaying virucidal effects. These findings suggest compound **6** does not directly inactivate viral particles as demonstrated by the absence of virucidal activity. Furthermore, derivative **6** is unlikely to interfere with viral attachment or adsorption and more likely acts during a subsequent stage of the viral replication cycle.

Given the limited number of 4-amino-7-chloroquinoline derivatives evaluated, the present study does not allow definitive structure–activity relationship conclusions to be drawn. However, the analysis of this small series suggests that the presence of a cyclohexyl moiety may be associated with increased antiviral activity within this chemical class. This preliminary observation provides a rationale for expanding the chemical series and systematically investigating structural modifications of the quinoline scaffold to further improve antiviral activity and selectivity.

Future studies should focus on elucidating the precise molecular mechanism of action of compound **6**, including its potential interaction with viral or host targets. In addition, in vivo evaluations, pharmacokinetic profiling, and toxicity studies, will be necessary to assess the potential of this scaffold for further antiviral development. The integration of in silico approaches and structure-based design may further support the rational development of more potent and selective 4-amino-7-chloroquinoline derivatives.

Overall, this work provides a foundation for the continued exploration of quinoline derivatives as candidate antiviral agents and contributes to ongoing efforts to develop effective therapeutic strategies against CHIKV and other medically relevant arboviruses.

## Supplementary Information

Below is the link to the electronic supplementary material.

Supplementary Material 1

## Data Availability

All relevant data supporting the findings of this study are included in the article and its supplementary materials.
