# Supplementary material for: Identification of 4-Amino-7-chloroquinoline Derivative with In Vitro Activity Against Chikungunya Virus
Source: Curr Microbiol. 2026 Aug 22;83(10):523. doi: 10.1007/s00284-026-05133-4 (PMC13499868; doi:10.1007/s00284-026-05133-4)
Supplement: Supplementary file 1 [file 284_2026_5133_MOESM1_ESM.docx]

**Identification of 4-Amino-7-chloroquinoline Derivative with In Vitro Activity Against Chikungunya Virus**

Adriana Cotta Cardoso Reis¹, Camila Portruneli¹, Lívia da Cunha Agostini^2^, Camila Rezende Minelli^3^, Luana Neves Dias^3^, Adalberto José de Lima^3^, Breno de Mello Silva⁴, Cíntia Lopes de Brito Magalhães⁴, Glenda Nicioli da Silva^2^, Guilherme Rocha Pereira^3^, Geraldo Célio Brandão¹*

¹ Federal University of Ouro Preto, Pharmacy School, Department of Pharmacy, Ouro Preto, MG, Brazil

² Federal University of Ouro Preto, Pharmacy School, Department of Clinical Analyses, Ouro Preto, MG, Brazil

^3^ Pontifical Catholic University of Minas Gerais, Department of Physics and Chemistry, Belo Horizonte, MG, Brazil

⁴ Federal University of Ouro Preto, Institute of Exact and Biological Sciences, Department of Biological Science, Ouro Preto, MG, Brazil

*Corresponding Author: Geraldo Célio Brandão, Federal University of Ouro Preto, Pharmacy School, Department of Pharmacy, Morro do Cruzeiro campus, Bauxita, 35402-163, Ouro Preto, MG, Brazil. Email: [celiobrandao@ufop.edu.br](mailto:celiobrandao@ufop.edu.br)

**Synthesis approach**

The synthesis of the 4-amino-7-chloroquinoline derivatives began with an electrophilic aromatic substitution between 4,7-dichloroquinoline and 2-aminoethanol. In this reaction, the amino alcohol acts as a nucleophile, featuring a short two-carbon side chain that links its functional group to the chloroquinoline scaffold, imparting a degree of conformational flexibility to the quinoline system. This step yielded intermediate 2, which was isolated as a white crystalline solid.

Subsequently, the amino alcohol was functionalized by bromination of the terminal hydroxyl group to introduce a more efficient leaving group for the following bimolecular nucleophilic substitution (SN2) reaction. This transformation was achieved by adding hydrobromic acid in toluene, facilitating the substitution of the hydroxyl group with a bromine atom. The reaction was performed using a Dean–Stark apparatus to continuously remove the water formed, thereby shifting the equilibrium toward product formation. Upon completion, the reaction mixture was neutralized, and the resulting product, 2-(2-bromopropyl)-7-chloroquinolin-4-amine (intermediate 3), was isolated as a white solid and purified for subsequent steps.

To obtain the final derivatives, a series of SN2 reactions were carried out by reacting five different anilines with intermediate 3 in the presence of dimethyl sulfoxide (DMSO). After synthesis and purification, detailed characterization was performed using ^13C and ^1H Nuclear Magnetic Resonance spectroscopy (400 MHz), confirming the molecular structures and purity of the compounds.

**Chemical Characterization**

**Compound 4**

**Fig. 1S.** ^1^H Nuclear Magnetic Resonance (^1^H NMR) spectrum of N^1^-(7-chloroquinolin-4-yl)-N^2^-(2,6-dimethylphenyl)ethane-1,2-diamine

**^1^H NMR (400MHz, CDCl_3_) ẟ (ppm)** (Figure S1): 12.28-12.26 (d, 1H, H8); 11.78-11.76 (s, 1H, H4); 11.75-11.71 (d, 1H, H1); 11.31-11.27 (d, 1H, H2); 10.93-10.89 (d, 2H, H18-H20); 10.80-10.75 (t, 1H, H19); 10.34-10.31 (d, 1H, H9); 8.17-7.98 (s, 1H, H12); 7.46-7.41 (t, H, H13); 7.29-7.25 (t, 2H, H14); 6.26-6.15 (s, 6H, H22-H23); 5.18-5.16 (s, 1H, H15).

**Fig. 2S.** ^13^C Nuclear Magnetic Resonance (^13^C NMR) spectrum of N^1^-(7-chloroquinolin-4-yl)-N^2^-(2,6-dimethylphenyl)ethane-1,2-diamine

**^13^C NMR (100MHz, CDCl_3_) ẟ (ppm)** (Figure S2): 154.93 (C5); 154.57 (C8); 151.62 (C10); 148.64 (C16); 139.59 (C3); 133.69 (C17); 133.00 (C21); 130.70 (C20); 129.55 (C18); 126.66 (C2); 125.99 (C4); 121.13 (C19); 102.61 (C1); 81.16 (C6); 52.76 (C9); 50.15 (C14); 47.32 (C13); 22.16 (C22-C23).

**Fig. 3S.** High-resolution mass spectrometry (HRMS-ESI) spectrum of N^1^-(7-chloroquinolin-4-yl)-N^2^-(2,6-dimethylphenyl)ethane-1,2-diamine. **HRMS-ESI:** *m/z* calcd. for C_19_H_20_ClN_3_ (M + H)^+^: 326.1379 found: 326.1416.

**Compound 5**

**Fig. 4S.**^1^H Nuclear Magnetic Resonance (^1^H NMR) spectru of N^1^-(4-chlorophenyl)-N^2^-(7-chloroquinolin-4-yl)ethane-1,2-diamine

**^1^H NMR (400MHz, CDCl_3_) ẟ (ppm)** (Figure S4): 12.30-12.28 (d, 1H, H8); 11.77-11.76 (d, 1H, H3); 11.75- 11.73 ( d, 1H, H6);11.28-11.25 (dd, 1H, H1); 11.05-10.50(m, 4H, H18, H20, H17, H21); 10.35-10.33 (d, 1H, H9); 7.62 (s, 1H, H12); 7.49-7.37 (m, 4H, H13, H14); 6.09 (s, 1H, H15)

**Fig. 5S.**^13^C Nuclear Magnetic Resonance (^13^C NMR) spectrum of N^1^-(4-chlorophenyl)-N^2^-(7-chloroquinolin-4-yl)ethane-1,2-diamine

**^13^C NMR (100MHz, CDCl_3_) ẟ (ppm)** (Figure S5): 155.4(C16); 154.6(C4); 152.3(C10); 150.5(C8); 133.1(C2); 131.2(C18); 129.39(C20); 118.0(C19); 102.7(C1); 81.45(C3); 81.12(C6); 80.8(C5); 5.3(C17); 53.0(C21); 52.9(C9); 52.6(C13); 52.4 (C14).

**Compound 6**

**Fig. 6S.**^1^H Nuclear Magnetic Resonance (^1^H NMR) spectrum of N^1^-(7-chloroquinolin-4-yl)-N^2^-cyclohexylethane-1,2-diamine

**^1^H NMR (400MHz, CDCl_3_) ẟ (ppm)** (Figure S6): 8.39-8.37 (d, 1H, H8); 8.12-8.09 (d, 1H, H4); 7.81-7.78 (d, 1H, H9); 7.44-7.42 (d, 2H, H1-H2); 6.59-6.57 (t, 1H, H12); 3.52-3.47 (m, 2H, H13); 3.00-2.94 (m, 2H, H14); 2.04-1.62 (m, 2H, H15-H16); 1.39-1.06 (m, 2H, H17-H21).

**Fig. 7S.**^13^C Nuclear Magnetic Resonance (^13^C NMR) spectrum of N^1^-(7-chloroquinolin-4-yl)-N^2^-cyclohexylethane-1,2-diamine

**^13^C NMR (100MHz, CDCl_3_) ẟ (ppm)** (Figure S7): 151.34 (C10); 151.11 (C5); 148.29 (C8); 134.98 (C3); 126.25 (C2); 124.69 (C4); 122.94 (C1); 117.43 (C6); 98.31 (C9); 56.55 (C16); 47.58 (C14-C17); 44.03 (C15); 42.24 (C21); 32.47 (C19); 25.75 (C18); 24.76 (C20).

**Fig. 8S.** High-resolution mass spectrometry (HRMS-ESI) spectrum of N^1^-(7-chloroquinolin-4-yl)-N^2^-cyclohexylethane-1,2-diamine **HRMS-ESI:** *m/z* calcd. for C_17_H_22_ClN_3_ (M + H)^+^: 304.1530 found: 304.1775.

**Compound 7**

**Fig. 9S.** ^1^H Nuclear Magnetic Resonance (^1^H NMR) spectrum of N^1^-(3 chlorophenyl)-N^2^-(7-chloroquinolin-4-yl)ethane-1,2-diamine

**^1^H NMR (400MHz, CDCl_3_) ẟ (ppm)** (Figure S9): 8.35-8.32 (d, 1H, H8); 8.05-8.04 (d, 1H, H3); 7.77 (d, 1H, H6); 7.39-7.37 (dd, 1H, H1); 6.78-6.67 (m, 3H, H18-H21-H19); 6.53-6.52 (d, 1H, H17); 6.53-6.52 (d, 1H, H9); 3.58-3.55 (t, 2H, H13); 3.44-3.40 (t, 2H, H14).

**Fig. 10S.**^13^C Nuclear Magnetic Resonance (^13^C NMR) spectrum of N^1^-(3 chlorophenyl)-N^2^-(7-chloroquinolin-4-yl)ethane-1,2-diamine

**^13^C NMR (100MHz, CDCl_3_) ẟ (ppm)** (Figure S10): 152.47 (C8); 151.43 (C3); 148.12 (C6); 142.30 (C1); 138.02 (C18); 124.67 (C21); 122.93 (C19); 117.36 (C9); 114.40 (C13); 93.33 (C14).

**Compound 8**

**Fig. 11S.** ^1^H Nuclear Magnetic Resonance (^1^H NMR) spectrum of N^1^-(7-chloroquinolin-4-yl)-N^2^-(4-fluorophenyl)ethane-1,2-diamine

**^1^H NMR (400MHz, CDCl_3_) ẟ (ppm)** (Figure S11): 8.57-8.55 (dd, 1H, H8); 8.17-8.14 (dd, 1H, H12); 7.95-7.93 (t, 1H, H20); 7.48-7.46 (t, 1H, H18); 7.45-7.44 (t, 1H, H17-H21); 6.38-6.80 (dd, 1H, H9); 4.66-4.60 (t, 1H, H1); 4.18-4.13 (t, 1H, H6-H3); 3.80-3.65 (s, 1H, H15); 2.36-2.33 (d, 2H, H13-H14).

**Fig. 12S.** ^13^C Nuclear Magnetic Resonance (^13^C NMR) spectrum of N^1^-(7-chloroquinolin-4-yl)-N^2^-(4-fluorophenyl)ethane-1,2-diamine

**^13^C NMR (100MHz, CDCl_3_) ẟ (ppm)** (Figure S12): 158.71 (C-19); 151.43 (C-4); 148.62 (C-10); 135.81 (C-16); 127.68 (C-8); 127.00 (C-2); 123.93 (C-1); 122.15 (C-6); 110.27 (C-3); 77.43 (C-5); 77.09 (C-18); 76.80 (C-20); 49.41 (C-17); 49.16 (C-21); 48.95 (C-9); 48.74 (C-13); 28,25 (C-14).
